# The effects of progesterones on blood lipids in hormone replacement therapy

**DOI:** 10.1186/s12944-017-0612-5

**Published:** 2017-11-21

**Authors:** Yifan Jiang, Weijie Tian

**Affiliations:** 1 Guizhou Provincial Center for Drug Reevaluation, Guiyang, Guizhou 550001 People’s Republic of China; 2 Department of Obstetrics and Gynecology, Guizhou Provincial People’s Hospital, NO.83, Zhongshan East Road, Guiyang, Guizhou 550002 People’s Republic of China

**Keywords:** Hormone replacement therapy, Progestogens, Lipids

## Abstract

The safety of progestogens as a class has drawn much attention after the publication of data from the Women’s Health Initiative (WHI) trial, particularly with respect to cardiovascular disease. Depending on the chemical structure, pharmacokinetics, receptor affinity and potency of action, progestogens have a divergent range of properties that may translate to very different clinical effects. The purpose of this review is to describe the role of varied progestogens in hormone replacement therapy (HRT), especially focusing on blood lipids, which are the most important parameters for assessing cardiovascular disease risk.

## Background

There are several diverse psychological symptoms due to estrogen deficiency during menopause including hot flushes, bouts of sweating, poor memory, insomnia, mood swings, vaginal atrophy and, most importantly, increased risk of cardiovascular disease [1]. Cardiovascular disease has long been recognized as the leading cause of death in women, which the risk significantly increases after menopause [2]. Menopause heralds the onset of low levels of high-density lipoprotein cholesterol (HDL-C), high levels of total cholesterol (TC), low-density lipoprotein cholesterol (LDL-C), very-low-density lipoprotein cholesterol (VLDL-C), triglycerides (TG) and lipoprotein(a) (Lp(a)), which are strongly predictive of an increasing risk for cardiovascular disease [3].

Hormone replacement therapy (HRT) is based on the idea that treatment may alleviate bothersome menopausal symptoms [4]. Several lines of evidence indicated that HRT has a protective effect against cardiovascular disease [5]. One of the most favorable properties of estrogen is its physiological effect on lipoproteins, including its ability to decrease LDL-C and increase HDL-C levels [6, 7]. An increased level of HDL-C is a particularly strong predictor of a decreased risk for cardiovascular disease in women [8].

Progestogens have typically been added to HRT to stimulate the endometrium to enter the secretory phase and to protect against the development of endometrial hyperplasia and carcinoma [9]. Progestogens include both natural progesterone and a variety of synthetic compounds named progestins [10]. Over the past decades, HRT combined with progestogens was widely prescribed with the expectation of cardiovascular disease prevention. However, the safety of this combination has been questioned after the results of the Women’s Health Initiative (WHI) trial were published. The data showed that the estrogen/progestogen formulation, compared with estrogen alone, displayed higher risks for cardiovascular disease and cancer [11, 12]. However, it should be noted that the WHI trial studied only one progestogen at one dose. Notably, all progestogens are not equal and may have distinct actions. [13]. Generalizing the results from WHI trial to include other progestogens used in combination with HRT would be questionable. The aim of this study is to review the available evidence on how the different progestogens may affect blood lipids in HRT.

## Classification of progestogens

Progestogens are a class of steroid hormones that bind to and activate the progesterone receptor (PR). They have a progestational activity, meaning the capacity to induce the secretory endometrium and support gestation [14]. Progestogens encompass natural progesterone and synthetic progestins. The term progestogen has been used synonymously with other terms, such as progestagen, gestogen, gestagen, and progestin [15]. To avoid confusion in light of current practices, the North American Menopause Society has recommended that the term “progesterone” should be used when referring to natural progestogens and “progestin” referring to synthetic progestogens [16].

Progesterone is the only natural progestogen that is used therapeutically. It is a compound identical to that secreted by the human ovary after ovulation and by the placenta during pregnancy. Relatively recent advances have allowed progesterone crystals to be micronized, resulting in improved oral absorption.

Progestins are a variety of synthetic progestogens. Based on the time of synthesis, progestins can be grouped into generations as follows [17, 18]:

- First generation: noretynodrel, nortestosterone derivatives (that become active after conversion to norethisterone), medroxyprogesterone acetate (MPA), cyproterone acetate, chlormadinone acetate and megestrol acetate.
- Second generation: norgestrel and levonorgestrel.
- Third generation: levonorgestrel derivatives such as desogestrel, gestodene and norgestimate.
- Fourth generation: dienogest, drospirenone, nestorone, nomegestrol acetate and trimegestone. The fourth generation, which has been synthesized in the past 2 decades, may be defined as “new progestins”. The property of the fourth-generation progestins is that they have no androgenic or estrogenic actions and are closer in physiological activity to the hormone progesterone [19].

Based on the chemical structure, progestins can be classified into two groups: ① those structurally related to progesterone and ② those structurally related to testosterone. Progestins structurally related to progesterone are subclassified into pregnane and 19-norpregnane derivatives, depending on those with and without a methyl group at carbon-10. These derivatives are further classified as those that are acetylated and those that are not. Progestins structurally related to testosterone can be subdivided into those that contain an ethinyl group at carbon-17 and those that are nonethinylated. The ethinylated derivatives are further classified as those that have an estrane structure and those that have a 13-ethylgonane structure [20]. The classification of progestogens is shown in Table 1.

**Table 1:** Classification of progestogens

Classification	Progestogen
Natural	Progesterone
Synthetic	Progestins
	A. Structurally related to progesterone
	*1. Pregnane derivatives*
	a. acetylated (also called 17-hydroxyprogesterone derivatives): medroxyprogesterone acetate, megestrol acetate, cyproterone acetate, chlormadinone acetate
	b. nonacetylated: dydrogesterone, medrogestone
	*2. 19-norpregnane derivatives (also called 19-norprogesterone derivatives)*
	a. acetylated: nomegestrol acetate, nestorone
	b. nonacetylated: demegestone, trimegestone, promegestone
	B. Structurally related to testosterone (also called 19-nortestosterone derivatives)
	*1. ethinylated:*
	a. estranes: norethindrone (also called norethisterone), norethindrone acetate, norethynodrel, lynestrenol, ethynodiol diacetate
	b. 18-ethylgonanes: levonorgestrel, norgestrel, desogestrel, gestodene, norgestimate
	*2. nonethinylated*: dienogest, drospirenone

## Mechanism of action of progestogens

Progestogens exert their physiological and biological effects by binding to and activating the progesterone receptors (PR). In humans, two progestogen receptor (PR) proteins, PRA and PRB, have been identified [21]. In addition, some progestogens can also modulate gene transcription through other steroid receptors, such as the androgen receptor (AR), glucocorticoid receptor (GR) and mineralocorticoid receptor (MR). The relative binding affinity data from various progestogens has been reported by Kuhl et al. [22] and Schindler et al. [23].

Progestogens have different pharmacological effects based on their structure. 19-norprogesterone and retroprogesterone derivatives tend to be pure progestogens. 17α-hydroxyprogesterone derivatives tend to have antiandrogen and glucocorticoid properties. 17α-ethynyltestosterone and 19-nortestosterone derivatives tend to possess weak androgenic actions. Gestodene is a 19-nortestosterone derivative with antimineralocorticoid properties. 17α-spirolactone derivatives tend to have antimineralocorticoid and antiandrogen actions. Progesterone itself has potent antimineralocorticoid properties and very weak glucocorticoid actions [13]. Very small structural changes may account for these considerable differences in the effects of progestogens.

## What kind of progestogens are used in HRT?

Progestogen has been used for decades to oppose the effects of estrogen on the endometrium. However, the approval of progestogens for drug use is relatively recent. Currently, progestogens can be administered through many routes: oral (tablet, capsule, and liquid), transdermal (topical patch, gel, and cream), vaginal (gel), intrauterine device (IUD), sublingual, intramuscular injection, rectal suppository, and subcutaneous implant. Progestogens used for HRT vary markedly between countries. Table 2 lists the progestogens and formulations that are available in China and the United States.

**Table 2:** Progestogens used for HRT in China and the United States. (values are based on the CFDA and FDA databases)

Composition	dosage forms	Available dosages
Progestogen-only		
Progesterone	Soft Capsules	100, 200 mg
	vaginal gel	4%, 8% gel
Medroxyprogesterone Acetate	Oral tablet	2.5, 5, 10, 100, 500 mg
Norethisterone (norethindrone)^b^	Oral tablet	0.35 mg
Levonorgestrel	Implants, Tablets, Intrauterine device	1.5 mg, 36 mg, 52 mg
Norgestrel	Tablets	0.075 mg, 3 mg
Dydrogesterone^a^	Tablets	10 mg
Combined with estrogen		
estradiol + norethindrone acetate	Film, Extended release /Transdermal	50 μg/24 h; 0.14 mg/24 h; 50 μg/24 h; 0.25 mg/24 h; 10 mg E + 30 mg P
	Tablets/Oral	1 mg/0.5 mg; 0.5 mg/0.1 mg
estradiol + drospirenone	Tablets/Oral	0.5 mg/0.25 mg; 1 mg/0.5 mg; 1 mg/2 mg
estradiol + dydrogesterone^a^	Tablets/Oral	1 mg/10 mg
estradiol + cyproterone acetate^a^	Tablets/Oral	2 mg/1 mg
conjugated equine estrogens + medroxyprogesterone acetate^b^	Tablets/Oral	0.3 mg/1.5 mg; 0.45 mg/1.5 mg; 0.625 mg/2.5 mg; 0.625 mg/5 mg
ethinyl estradiol + norethindrone acetate^b^	Tablets/Oral	5 μg/1 mg; 25 μg/0.5 mg

Products not marked are available in both China and the United States

^a^Available only in China

^b^Available only in the United States

## Clinical effects of progestogens on blood lipids in HRT

### Progesterone

Some progestogens abrogate the beneficial effects of estrogens on lipid metabolism [24]. A few studies have found that progestogens with greater androgenicity have more impact to attenuate the putative benefits of estrogens compared to progestogens with lower androgenicity [6, 25, 26]. There is a hypothesis that attenuation of the favorable lipid profile induced by estrogen is related to the intrinsic androgenic activity of progestogens. Progesterone demonstrates a strong progestogenic and antiestrogenic activity in the endometrium and cervix but without any androgenic effects. Based on the hypothesis as mentioned above, progesterone is supposed to have a neutral effect on lipid metabolism. Some recent publications presenting results are in favor of this idea.

Two randomized studies examined the effect of progesterone alone on hormone levels in postmenopausal women [27, 28]. The first was a small trial with 20 healthy postmenopausal women for 6 weeks of treatment with micronized progesterone (100 mg/daily). The second one was a 3-month placebo-controlled trial of progesterone (300 mg/daily) with 133 healthy postmenopausal women. Both trials found that progesterone alone did not change most lipids levels, except for HDL-C in the second study. The author mentioned that the statistically significant decrease in HDL-C levels was not clinically important. Another study showed that progesterone alone could not alter VLDL-TG in postmenopausal women, which means it has no potential role in mediating TG concentration [29].

Several studies have permitted a comparison between the unopposed estrogens and estrogens plus progesterone. A retrospective study analyzed post-menopausal women receiving HRT for 10 years compared to parallel cohorts [30]. Regimens included were as follows: control group, transdermal estradiol group, transdermal estradiol group plus 200 mg/day micronized oral progesterone (sequential-cyclic), or transdermal estradiol plus 100 mg/day micronized oral progesterone (continuous-combined). The results showed that there were no significant differences in TG, TC, HDL-C and LDL-C levels observed between the estradiol group and estradiol-combined progesterone group. Another two studies assessed the effects of the short-term use of vaginal micronized progesterone added to non-oral estrogen (intranasal or transdermal) on the lipid profiles of postmenopausal women [31, 32]. The results indicated that micronized progesterone by vaginal route did not alter the favorable response of non-oral estrogen on the lipid profiles of the women. Similarly, a 3-year multicentered, randomized trial found that progesterone, when combined with oral conjugated equine estrogen, did not interfere with cholesterol, cholesterol subfractions or TG caused by oral estrogen [33]. The results from a clinical trial showed adding natural micronized progesterone may counterbalance the TG-increasing effect of conjugated equine estrogen (CEE), which the author also stated might be due to small sample size, and they recommended further study [34]. In light of these data, progesterone may not adversely influence the protective benefits of blood lipids induced by estrogens.

### Medroxyprogesterone acetate (MPA)

Starting with progesterone, the addition of a hydroxyl group at carbon-17 and a methyl group at carbon-6, MPA exhibits relatively high progestational activity and better bioavailability [20]. It acts as an agonist of the progesterone, androgen and glucocorticoid receptors but has negligible affinity for the estrogen receptor [13].The intrinsic activities of MPA in activating the PR and the AR have been reported to be at least equivalent to those of progesterone and dihydrotestosterone, indicating that it is a full agonist of these receptors [35]. MPA was the most commonly used progestin in the US until the results of the WHI trial indicated that conjugated equine estrogens (CEE) + MPA increased the risks of cardiovascular disease and thromboembolism compared with CEE alone [11]. In the postmenopausal estrogen/progestin interventions (PEPI) trial, the most favorable effect on HDL-C concentrations was observed in women taking unopposed estrogen. Adding MPA diminished many of the benefits of estrogen [33]. Consistent with the PEPI trial results, many other studies have also reported attenuating effects of MPA on blood lipids, especially HDL-C [36–41]. For example, Lobo et al. [36] evaluated the effects of oral CEE and MPA on cardiovascular disease risk factors in healthy postmenopausal women for one year. A dose-dependent decrease in TC and LDL-C induced by CEE was not affected by MPA. HDL-C levels were increased with all doses of CEE, and MPA attenuated this effect in a dose-dependent manner. In another trial [37], Kim et al. found that CEE alone increased HDL-C, but the combination of 5 or 10 mg/day MPA offset this effect. Manwaring et al. [39] conducted a crossover study focusing on type II diabetic postmenopausal women. The results indicated that the addition of MPA abolished the increase in HDL-C associated with CEE but did not significantly affect any other lipid measurements. This study added further confirmation to the attenuating effects of MPA on HDL-C in both healthy and type II diabetes postmenopausal women.

However, there are some trials that indicated that MPA did not change the lipoprotein profile. Sai et al. [42] examined the effect of CEE alone or in combination with MPA on the lipid profiles of elderly postmenopausal women. The results showed that CEE with or without MPA lowered serum LDL-C, increased HDL-C and TG. Espeland et al. [43] found that estrogen combined with MPA also produced consistent and sustained reductions in plasma Lp(a) concentrations. A study from Wolfe et al. [44] found that continuous administration of low-dose MPA (2.5 mg/day) preserved the beneficial effects of CEE on plasma lipoproteins. A significant increase in HDL cholesterol and a significant decrease in LDL cholesterol were found in this study. AinMelk et al. [45] evaluated the effects of two oral continuous combined regimens of HRT, a conjugated estrogen with MPA, and an estrone sulfate with MPA during a 104-week period on lipid metabolism in 59 postmenopausal women. TC and LDL-C levels decreased significantly. Increasing HDL-C and TG levels were noted but without significance. The author thought continuous MPA does not negate the beneficial effects of HRT on lipid metabolism. Tufekci et al. [46] found that MPA, whether used continuously or sequentially, does not oppose the beneficial effects of transdermal 17β-estradiol on the lipoprotein profile. The same result was also evident in another study [47]. Such discrepancies between these results may be due to study design, cohort chosen for the study, estrogen dosage or size of the subjects. In short, the concept that MPA reverses the benefits of estrogen on lipid parameters is still controversial.

### Dydrogesterone

Dydrogesterone is an orally active progestogen that is chemically and biologically very similar to natural progesterone [48]. It has an additional double bond between carbon-6 and carbon-7. As a consequence, dydrogesterone has potent progestogenic and antiestrogenic activity but without any estrogenic, androgenic, antiandrogenic or glucocorticoid activity [49]. In 1994, Voetberg et al. [50] assessed the change in the lipid profiles of 165 healthy postmenopausal women treated with 2 mg 17β-estradiol continuously combined with four doses (2.5, 5, 10 or 15 mg) of dydrogesterone for 6 months. With all four dosages of dydrogesterone, the lipid profiles (TC, HDL-C, LDL-C and apolipoproteins) improved significantly. The data suggested that continuously applied dydrogesterone in combined-HRT did not diminish the beneficial effects on lipid metabolism induced by estrogens. Similar results were observed in other studies [51–59]. Overall, when given in combination with estrogen, dydrogesterone does not have a large impact on estrogen effects. This is especially true for an increase in HDL-C and a reduction in LDL-C caused by estrogen, as these effects persisted in combination with dydrogesterone.

### Norethisterone acetate (NETA)

Norethisterone acetate (NETA), also known as norethindrone acetate in the United States, is a steroidal progestin of the 19-nortestosterone group [60]. After oral ingestion, NETA is rapidly hydrolyzed into norethisterone (NET) by esterases during intestinal and first-pass hepatic metabolism [61]. Both NETA and NET compounds are structurally related to testosterone and are characterized by the presence of an ethinyl group at carbon-17 and the absence of a methyl group at carbon-10. NETA exhibits strong tissue-specific progestogenic, estrogenic, antiestrogenic and androgenic effects [20].

A 5-year controlled study showed that estradiol, combined with NETA, decreased TC and LDL-C levels by 20%, and HDL-C was virtually unchanged [62]. In another study, Munk et al. conducted a 2-year study to examine the effects of 17β-estradiol and NETA on plasma lipoprotein levels. The results showed that HDL-C levels were reduced in the combined estradiol and NATA group, whereas the reductions in LDL-C concentrations remained [63]. Similar results have been found by other investigators [64–67]. Furthermore, Kwok et al. [68] examined the effect of MPA and two other progestogens, the less androgenic desogestrel and the more androgenic norethisterone (NET), on cardiovascular risk factors when combined with estrogen therapy. They found that all the progestogens tested in the study significantly reduced HDL-C levels. NET was associated with the greatest reduction in HDL-C and in TG. The evidence from these studies indicated that NETA is a unique progestin that exerts both estrogenic and androgenic properties. It reverses the beneficial effect on HDL but also favorably influences the TG, VLDL, LDL subfraction profiles and lipoprotein(a).

### Nomegestrol acetate

Nomegestrol acetate is considered to be a “new” progestin. It is formed by adding a double bond between carbon-6 and carbon −7 of the hydroxyprogesterone skeleton and deleting the CH3 radical at carbon-19 [69]. It acts as a selective, high-affinity full agonist of the progesterone receptor, which confers a higher progestational potency than MPA [70]. Consistent with this, nomegestrol acetate is a potent antigonadotropin but is completely devoid of androgenic effects on male accessory sex organs [71].

In experiments testing the effects of nomegestrol acetate on hormonal, metabolic and hemostatic parameters in 36 premenopausal women, Basdevant et al. [72] found no effects of nomegestrol acetate alone on HDL-C, LDL-C, apolipoprotein B, fibrinogen or plasminogen levels. TG and apolipoprotein A1 levels decreased significantly. In a double-blind, randomized, prospective, three-cycle study of 57 non-hysterectomized women with natural menopause, the effects of estradiol plus nomegestrol acetate (1.5 mg or 3.75 mg) or placebo on cardiovascular risk factors were evaluated. Both the high- and low-dose estradiol plus nomegestrol acetate combinations had favorable effects on plasma lipids and lipoproteins, including significant reductions in LDL-C and lipoprotein(a). The increase in HDL-C was not statistically significant [73]. These results are in agreement with other studies in which no clinically relevant changes were seen in TC, HDL-C, LDL-C, TG [74] or TC; additionally, the LDL levels were significantly decreased and the LDL:HDL ratio was reduced by 10% [75]. Altogether, these results suggest that nomegestrol acetate, a 19-norprogesterone derivative with potent progestational activity and no androgenicity, does not counteract the effect of estrogen on lipoprotein concentrations.

### Drospirenone

Drospirenone is a testosterone- and spironolactone-derived molecule. It has the basic 19-carbon chemical structure of its parent compound, androstane [18]. Drospirenone is a novel progestin similar to natural progesterone [19], as it combines potent progestogenic and antiandrogenic activities. In addition, it has antimineralocorticoid properties not found in most synthetic progestins [76].

In a multicentered, double-blind, randomized study, 1142 postmenopausal women were treated with estradiol alone or estradiol plus 0.5, 1.0, 2.0, or 3.0 mg of drospirenone. The results showed that TC and LDL-C values significantly decreased, HDL-C significantly increased in the estradiol/drospirenone group [77]. These data are consistent with the results of other studies [78, 79] which showed drospirenone has beneficial effects on blood lipids. However, Paoletti et al. reported that lipoprotein parameters and TG did not vary in the estradiol/drospirenone group. Further studies of longer durations are required before conclusions can be reached regarding the putative favorable effects of drospirenone on lipids profiles in HRT.

## Conclusion

For many decades, it is a common misconception that all progestogens have similar mechanisms of action and produce the same effects. There is an increasing body of data showing that progesterones are not all the same in modifying blood lipids induced by estrogens (Table 3). Some progestins with androgenic properties diminish the beneficial effects of estrogens on lipoprotein metabolism, whereas progesterone and some 19-norprogesterone derivatives do not adversely influence these protective effects of estrogens. Therefore, it is inappropriate to generalize the various effects of progesterones. The properties of each progestogen should be carefully evaluated on an individual basis to determine its utility in HRT. Currently, no long-term clinical data from trials comparable to the WHI study are available for other HRT combinations. Such studies are needed to confirm if the observed changes in blood lipids are large enough to be of clinical significance in perimenopausal and postmenopausal women.

**Table 3:** Specific characteristics of the progestogens in HRT

Progestogens	Notable characteristic in HRT
Progesterone	Have neutral effect
MPA	May have attenuating effects but still controversial
Dydrogesterone	Have neutral effect
NETA	Reverses the beneficial effect on HDL but also favorably influences the TG, VLDL, LDL
Nomegestrol acetate	Have neutral effect
Drospirenone	Have favorable effects

## Abbreviations

AR: Androgen receptor
CEE: Conjugated equine estrogens
GR: Glucocorticoid receptor
HDL-C: High-density lipoprotein cholesterol
HRT: Hormone replacement therapy
LDL-C: Low-density lipoprotein cholesterol
Lp(a): Lipoprotein(a)
MPA: Medroxyprogesterone acetate
MR: Mineralocorticoid receptor
NETA: Norethisterone acetate
PEPI: Postmenopausal estrogen/progestin interventions
PR: Progesterone receptor
TC: Total cholesterol
TG: Triglycerides
VLDL-C: Very-low-density lipoprotein cholesterol
WHI: Women’s Health Initiative
